# Severe Pilon Fractures: The Role of Quality of Reduction in Clinical and Functional Outcomes

**DOI:** 10.7759/cureus.65245

**Published:** 2024-07-24

**Authors:** Konstantinos Tilkeridis, Efthymios Iliopoulos, Simon Wall, George Kiziridis, Arshad Khaleel

**Affiliations:** 1 Orthopaedic Surgery, University General Hospital of Alexandroupolis, Democritus University of Thrace, Alexandroupolis, GRC; 2 Orthopaedics, University General Hospital of Alexandroupolis, Democritus University of Thrace, Alexandroupolis, GRC; 3 Trauma and Orthopaedics, Ashford and St. Peter's Hospitals NHS Trust, Chertsey, GBR

**Keywords:** ankle fractures, illizarov, fracture reduction, type iii, pilon fracture

## Abstract

Introduction

The purpose of the current study is to present the outcome of closed reduction and stabilization using an Illizarov ring fixator in severe pilon fractures and to investigate the correlation between reduction quality and clinical and functional outcomes.

Materials and methods

Thirty-three type III tibial plafond fractures, which had been treated with this method, were retrospectively analysed. Quality of reduction was classified according to the Teeny & Wiss (TW) criteria. Clinical and functional assessment was carried out using the Ovadia & Beals (OB) and Olerud & Molander (OM) scores.

Results

All fractures were successfully united. The mean time in the fixator was 6.3 months, and the mean follow-up was 50 months after frame removal. There were no major infections. There was no significant relationship between TW and OM (r=-0.34, p=0.13), TW and OB (r=0.35, p=0.23), neither Delay (from injury until surgery) and OM (r=-0.03, p=0.28), and Delay and OB (r=0.30, p=0.31).

Conclusions

The present study demonstrates that the major problems of open reduction and internal fixation of pilon type III fractures can be avoided by a non-invasive approach to the treatment of these fractures. The articular surface can be reconstituted with olive-tip wires and small fragment washers, early ligamentotaxis and fracture stabilization with the Ilizarov ring fixator. These simple steps could lead safely to union and a good clinical and functional outcome.

## Introduction

Pilon fractures are intra-articular fractures of the distal tibia with variable proximal extension. The injury is caused by vertical loading, driving the talus into the tibial plafond, causing bony comminution and irreversible articular cartilage damage. This typically occurs after a fall from a height or road traffic accident. The foot position and rate of loading influence the final injury pattern [1].

Pilon fractures have been classified by Ruedi and Allgower into three types according to the fracture displacement and comminution; with type III representing greater fracture displacement and comminution and associated soft tissue trauma [1]. The majority of the literature assesses type I and II fractures alongside type III injuries. However, type III injuries have been shown to have a consistently inferior outcome when compared to all methods of management, including internal and external fixation [2-5].

Watson et al. showed that the severity of the fracture was directly related to the grade of soft tissue damage in closed injuries [3]. These severe injuries are often open, and whether open or closed, have the potential for a high rate of wound complications. In recent years, many authors have consistently advised the need to minimise further soft tissue trauma during operative reduction and stabilisation of these injuries [2,3,5,6]. Thus, some authors propose initially external fixation followed by delayed open reduction and internal fixation (ORIF) [7] while others propose external fixation alone or combined with limited open reduction, arthroscopic-assisted reduction [5], limited internal fixation and plating of the fibula [8]. Many different methods of external fixation have been advocated in the management of these injuries and are often combined with limited open reduction, arthroscopic assisted reduction [5], limited internal fixation and plating of the fibula. Although, there are several clinical trials in the literature presenting outcomes after pilon fracture treatment, or comparing results of different types of management, very few of them investigate the correlation between quality of reduction, clinical objective results and subjective functional assessment.

The purpose of this study was to assess the outcomes of type III pilon fractures managed solely by closed indirect reduction and stabilisation with the Ilizarov ring fixator and to investigate their association with the parameters mentioned above.

## Materials and methods

**Figure 1:**
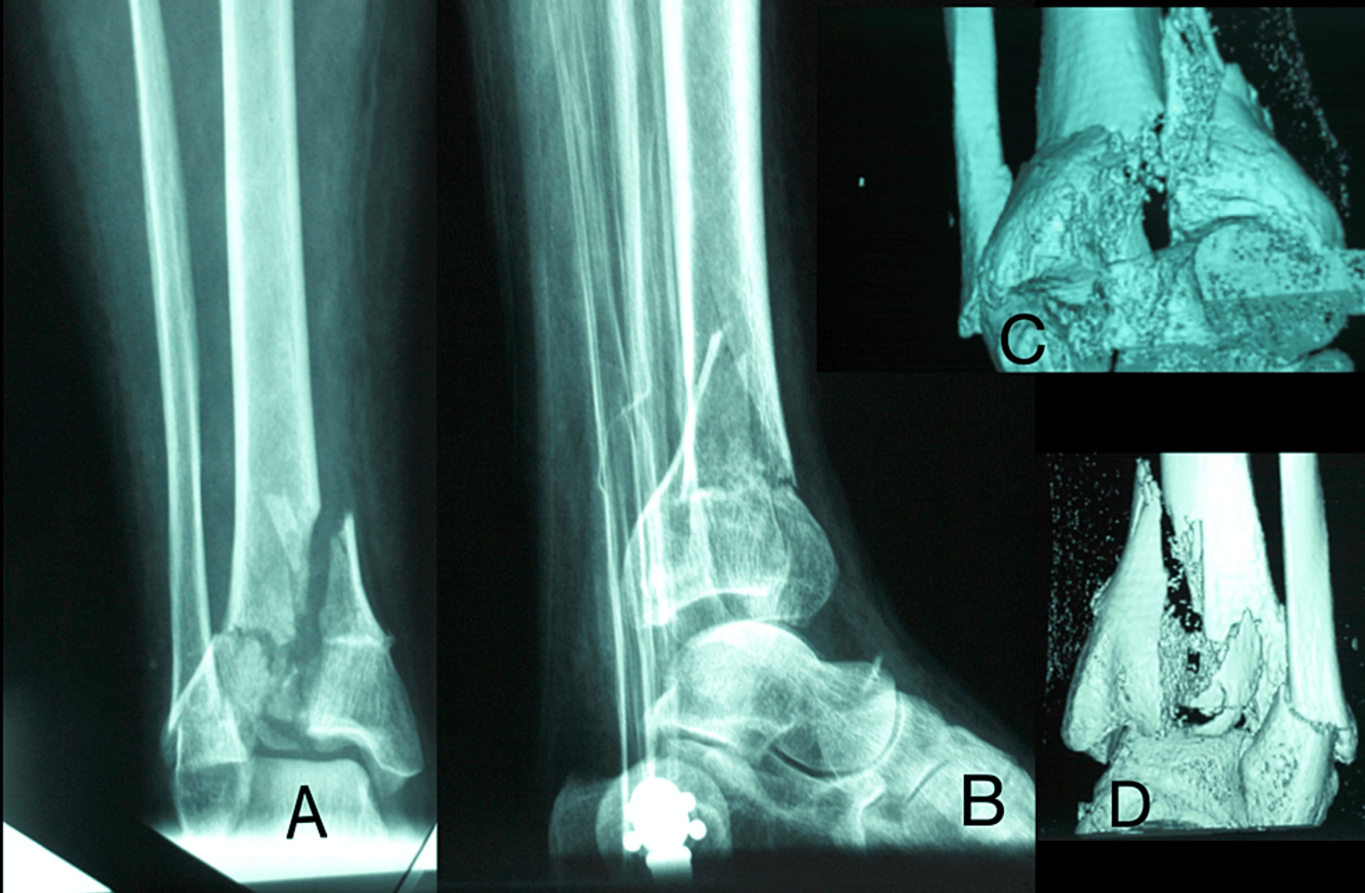
Anteroposterior (A), lateral (B) radiograph and 3D reconstruction CT scan (C,D) demonstrating the explosive nature of the severe pilon injury.

From January 1995 to May 2005, 44 type III pilon fractures in 42 patients were identified from the trauma database of our hospital. They were all treated primarily with the Ilizarov ring fixator. Patients originated from two sources, either from the local Accident and Emergency (A&E) department or as a tertiary referral from surrounding units. All fractures were identified on a plain anteroposterior and lateral radiograph in the A&E department, whereas all patients underwent a computed tomography scan with 3D reconstruction (Figure 1). During full assessment and resuscitation of the patient, grossly displaced fractures were reduced and held in a plaster back-slab or a calcaneal traction pin was applied. Open injuries were photographed and swabbed, and patients were covered for tetanus and given broad-spectrum antibiotics. They were then taken to the theatre for urgent debridement and either temporary or definitive fixation.

Immediate definitive fixation was dependent on the patient initially presenting to the specialist centre and a surgeon with the necessary skills being on-call. Patients from other centres were transferred at the earliest opportunity, but this often incurred a delay and made up the majority of the average 5.6-day delay between injury and definitive surgery (range 0-19).

Data were collected by two of the authors (SW and KT) and included age at the time of injury, the cause of the injury, whether the fracture was open or closed, delay in definitive treatment, average hospital stay, length of time in the frame and any complications during treatment. Specific data collection included the following.

Reduction of the Fracture

The quality of the reduction of the fracture was assessed from either the final intraoperative or the immediate postoperative radiographs. This was classified as anatomic, good, fair or poor on the basis of a numerical summary score according to the criteria proposed by Ovadia and Beals [9] and modified by Teeny and Wiss (T&W) [2]. Especially, the sum of eight parameters, measured in millimetres, and scored according to a structured system represented the numerical rating of the quality of reduction. Those measurements were displacement of the lateral, medial and posterior malleolus, mortise and fibular widening, talar tilt and shift and articular gap. Finally, a total score of 8 points represented Anatomic, 9-11 was Good, 12-15 was Fair, while more than 15 presented as Poor quality of reduction.

Clinical Assessment

Clinical assessment was carried out at every follow-up using the Ovadia and Beals (O&B) [9] objective ankle evaluation. The most present results were included in the statistical analysis. Subjective assessment utilised with the Olerud and Molander (O&M) ankle score [10], which is a patient-reported outcome measure (PROM), with scoring scales of pain, stiffness, swelling and function. This score was found to correlate well with a linear analogue scale, limitation in range of motion while loading and the presence of osteoarthritis or dislocation in radiographs, which are considered to represent the results after ankle fractures [10].

Patients were also asked to complete the World Health Organization Quality of Life Brief Version (WHOQOL-BREF) health questionnaire. This allows comparison with the mean for the population, according to published norms. All patients within one standard deviation of the norm are accepted as within the normal population [11].

Surgical Technique

**Figure 2:**
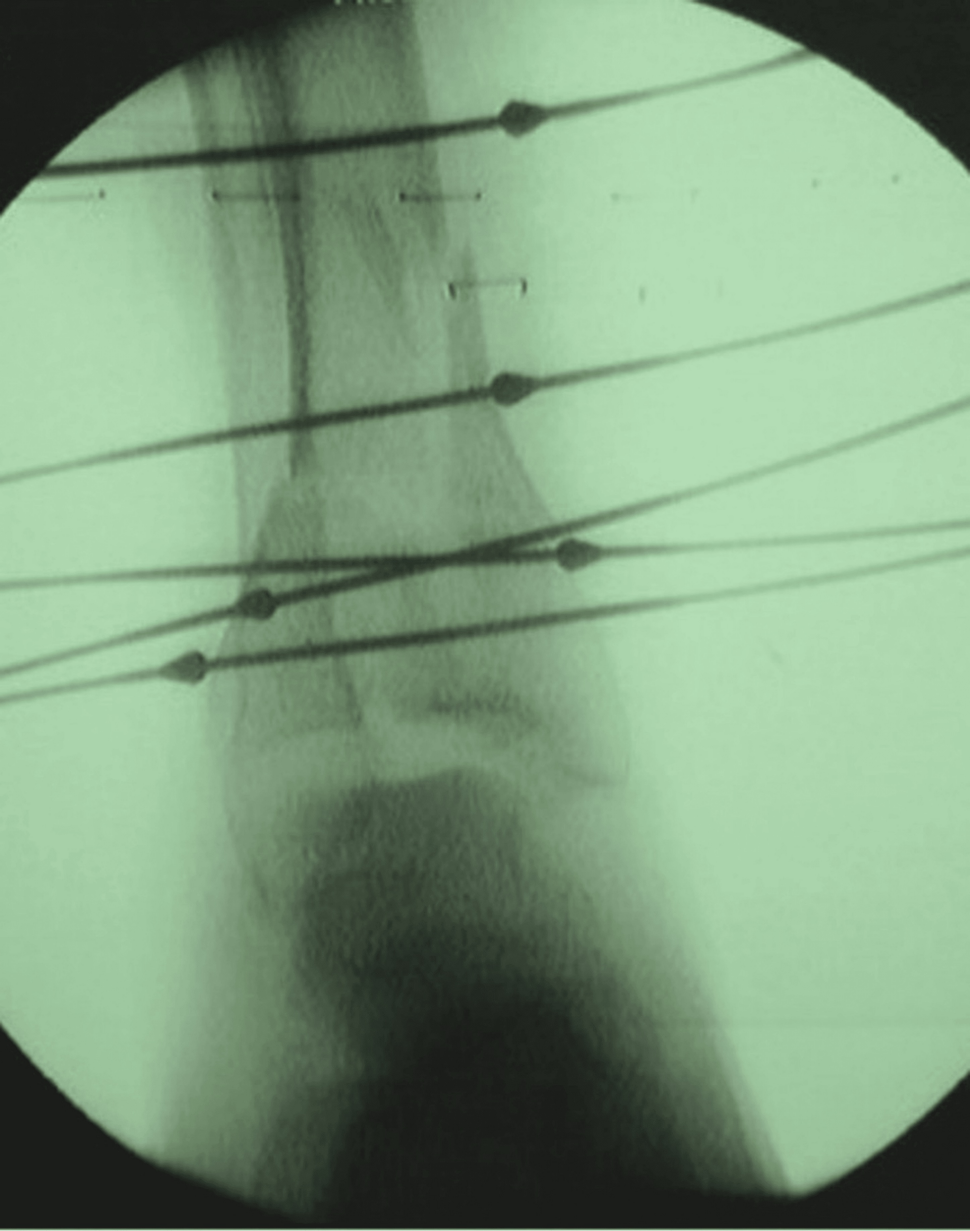
Anteroposterior radiograph of a pilon fracture and its reconstruction using the Ilizarov method

**Figure 3:**
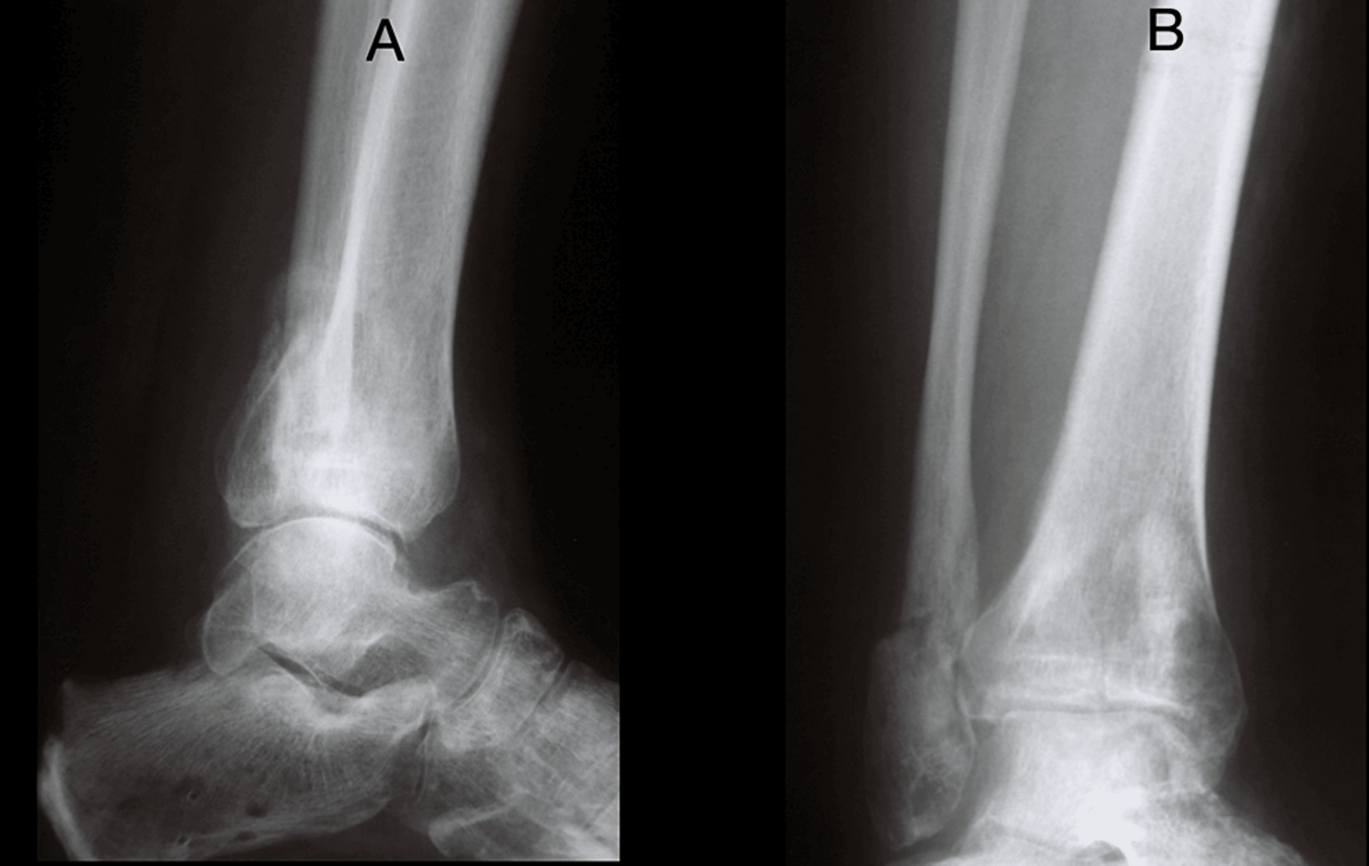
Lateral (A) and anteroposterior (B) views of a treated pilon fracture using the Ilizarov frame during follow-up

The frames consisted of three rings with an additional footpiece. After the insertion of proximal and distal reference wires, traction was applied between the proximal tibia and the footpiece in order to achieve indirect reduction with ligamentotaxis. Afterwards, olive wires were used for fracture fragment reduction and small fragment washers where necessary (Figure 2). In order to achieve compression at the fracture site, tensioning through olive wires was applied, and when appropriate, compression through the frame was achieved. After the frame assembly, the footpiece was detached and an evaluation of the fixation stability and ankle joint stability was performed. If the construct/fracture was evaluated as stable without the foot piece, the footpiece was removed at the end of the surgery. Patients were mobilised immediately post-op and were allowed to weight-bear fully, but this was often delayed by discomfort and/or uncertainty by the patient. Post-operatively, the patient was educated about frame care. Pin site care was performed every three days using chlorhexidine. The frames were removed when clinical and radiological union were assessed to have been achieved, and patients were mobilised immediately in an aircast boot (Figure 3).

Statistical analysis

In order to find out any relationships between the measured parameters, we have performed correlation coefficient (r) and analysis of variance (ANOVA) between the following pairs: T&W vs. O&M, T&W vs. O&B, Delay (from injury until surgery) vs. O&M, and Delay vs. O&B. SPSS (v13.0, Chigaco, Illinois) was used for the statistical evaluation. p<0.05 was considered significant.

## Results

From the 42 included patients, the follow-up review revealed that one patient had died, another underwent primary ankle arthrodesis for bilateral injury, four failed to attend follow-up, but confirmed good general function or return to work, and four patients were lost to follow-up. This left 34 ankles in 32 patients who were reviewed in a special follow-up clinic. There were 12 females and 20 males. Follow-up averaged 34 months with a range of 6 months to 10 years 6 months.

The fractures united in all patients. The mean time in the fixator was 6.3 months (range 3-10 months). The mean follow-up was 50 months after frame removal (12-84 months range). Fracture fixation after the evaluation at the end of surgery was stable without the use of a footpiece so this was removed in six cases. In 28 cases, it was kept on for the duration of the treatment. There were no major or deep infections. Pin tract infections occurred in most of the cases but responded to rest and oral antibiotics. There were 2 unplanned operations: excision of a ring sequestrum in one patient performed 15 months after the injury and excision of osteophytes from the ankle joint in another at 3 years.

The Olerud and Molander ankle score ranged from 35-95 with a mean of 74 [10]. Eight patients had good to excellent scores (>70); 5 had fair scores (60-69) and 21 patients had poor scores (<59) (Table 1). Radiological assessment showed anatomic reduction was obtained in 4 ankles, a good reduction in 10, and fair or poor reduction in the rest of the 18 patients (Table 1). According to Ovadia and Beals score [9], 6 patients had excellent scores, 19 patients had good scores, and 9 patients had fair and poor scores (Table 1). There was one malunion - the fracture uniting in 15 degrees of varus - he had the only poor clinical score. The same patient had the longest wait of 17 days between injury and frame application.

**Table 1:** Quality of reduction, functional and clinical score of all fractures. Data are presented as % (N) in the table. p<0.05 considered significant.

	Tynee &Wiss	Olerud & Molander	Ovadia & Beals
Excellent	11.8% (4)	11.8% (4)	17.6% (6)
Good	29.4% (10)	11.8% (4)	55.8% (19)
Fair	32.35% (13)	14.7% (5)	11.8% (4)
Poor	20.5% (7)	61.8% (21)	14.7% (5)

Ten patients had radiological evidence of osteoarthrosis. Two of these had subchondral sclerosis alone. In the remaining eight, there was sclerosis as well as joint space narrowing; periarticular osteophytes developed in two of these patients later.

The WHOQOL-BREF scores revealed a mean physical component score of 55 (range 36-79), 19 being within 1 standard deviation from the norm based on mean scores (M=73.5 SD=18.1) [11]. Thus 15 patients were outside results expected for a normal population, which is not surprising in a group with such a severe injury. The mean mental component score was 68 (range 48-77), 30 being within 1 standard deviation of the norm [11].

**Figure 4:**
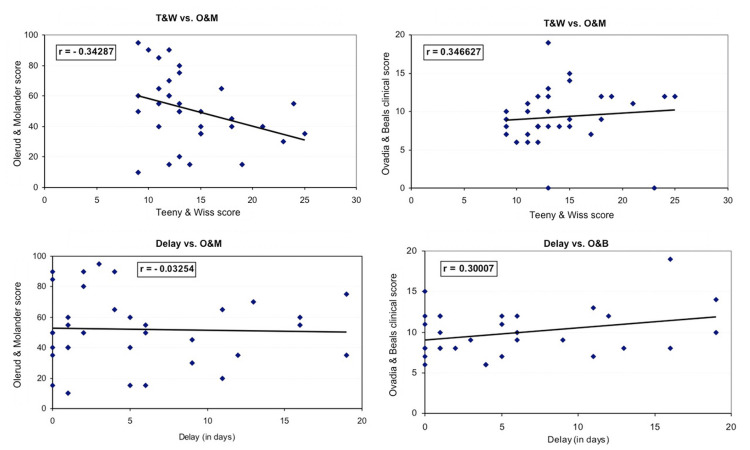
Correlations Correlation of the quality of the reduction (Teeny & Wiss score) with the subjective and objective clinical scores (Olerud & Molander, Ovadia & Beals scores). Correlation of the delay of the surgery with the above clinical and functional scores.

Finally, there was no significant relationship between T&W vs. O&M (r=-0.34, p=0.13), T&W vs. O&B (r=0.35, p=0.23), Delay (from injury until surgery) vs. O&M (r=-0.03, p=0.28), and Delay vs. O&B (r=0.30, p=0.31) (Figure 4).

## Discussion

The most important result in our series was the non-significant association of the quality of reduction with the clinical assessment and subjective functional scores. The values of the correlation coefficient between the above parameters were weak to moderate without statistical significance. We consider that the key elements to achieve a good outcome in these severe injuries are restoring the axial alignment using ligamentotaxis and stabilizing the fracture using circular fixators, like the Ilizarov device, in addition to respecting the soft tissue envelope and the blood supply of the intraarticular fracture fragments. The poor outcome seems to be the result of the initial cartilage damage at the time of the injury, irrespective of the often good or even anatomic joint reconstruction [12]. 

Articular congruity affects the functional outcome [1,2,13,14], but the perfect anatomical reduction cannot promise excellent clinical results. Teeny and Wiss in 30 type III pilon fractures treated with internal fixation found that 4 (10%) fractures required late ankle arthrodesis despite good articular reduction, whereas 7 (32%) required arthrodesis when the reduction was poor [2]. In a retrospective review of 14 Ruedi type III fractures treated with cross-ankle circular external fixation, Kapukaya et al. reported excellent and good outcomes, despite the anatomical reduction of the articular surface in only 10 of them [5]. On the other hand, Lovisetti et al. presented clinical outcomes in a series of severe pilon fractures, which have been less favourable than others, despite the quality of reduction [15]. Their series had 30 cases of pilon fractures treated with a ring fixator combined with minimal internal fixation. Twenty-eight of them had anatomic or good reduction, but only 14 of them reported excellent or good clinical results. The question is whether we should aim to restore the articular congruity at any cost or whether other parameters are more important.

Whilst justifying the importance of restoring articular congruity, Teeny and Wiss demonstrated the consequence of open surgery through badly contused and swollen soft tissues [2]. Following open surgery, the incidence of skin slough and wound dehiscence more than doubled while the rate of deep infection increased six-fold in type III fractures compared to type I and II pilon fractures.

The ideal management of pilon fractures requires articular reconstruction and fracture stabilization whilst avoiding any further trauma to the badly damaged soft tissues, but Ruedi type III or Tscherne type 3 dictates a two-step approach or direct external fixation using circular devices [12]. This can be achieved using the Ilizarov circular frame. In 8 type B and 13 type C pilon fractures, Vidyadhara and Rao used only Illizarov ring fixation and limited internal reduction of the articular line if needed [16].

Although 8 of the patients developed arthritis in two years of follow-up, 76% of them had excellent functional results. In our study, no invasive procedures were carried out. Exposure of the fracture site and dissection of the compromised soft tissues was avoided. Articular congruity and bony alignment were restored indirectly using ligamentotaxis with the Ilizarov frame. The fragments were held in position by olive tip wires and small fragment washers where necessary. Good radiological articular reduction was restored in 16 out of the 34 patients. Bony union was achieved in all patients. All major wound complications, such as sloughing, dehiscence and deep infections, were avoided. Bone grafting was not required in any of the patients. The ring fixator provides a stable construct, thereby preventing any further soft tissue damage and allowing immediate weight bearing. Minor infections related to the pin sites invariably occurred but they all responded to rest and antibiotics [17]. The functional outcome of our series with 25 out of the 34 ankles having good or excellent results compares well with those of the published series [17-19].

Previous studies associate the outcome of type III pilon fractures with the extent of the initial soft tissue injury and with the subsequent treatment, in particular, the management of the soft tissues during fracture treatment [2,3,7,14,20,21]. The long-term outcome of pilon fractures is also affected by fracture patterns [22]. Whilst low-energy fractures can be successfully treated with internal fixation [1,23], the treatment of severe type III pilon fractures using similar techniques is associated with a high rate of both early and late complications. The incidence of these complications includes skin necrosis and wound dehiscence (20%), deep infection and osteomyelitis (17%), malunion (42%), non-union (25%) and post-traumatic arthrosis (50%). The need for secondary salvage procedures after this injury has been reported to be as high as 42%, including arthrodesis (32%) and even below-knee amputation (6%) [9,13,24-26].

The high rate of these unforgiving complications has been responsible for alternative approaches to the management of these high-energy pilon fractures, which involve immediate fracture stabilization with external fixation followed by delayed, open articular reconstruction [7,19,21,27-29]. Gehr et al. presented an alternative management of distal metaphyseal tibial and pilon fractures with percutaneous nailing of the fibula and distal tibia following joint reconstruction [4]. Eleven out of 15 patients had excellent and good objective and subjective results but only one year of follow-up. McDonald reported no deep infection, one delayed union and one malunion in 13 type III pilon fractures treated with an Ilizarov fixator and limited arthrotomy [28]. Similarly, Bonar reported no wound infection, skin slough or osteomyelitis when 12 type III fractures were treated with unilateral external fixation and limited internal fixation [29]. While the incidence of major complications is significantly reduced, there are still problems with this technique. Tornetta had one deep infection, one malunion and three pin track infections when combining internal and external fixation for pilon fractures [19]. Rampurada et al. reported two delayed unions, one non-union, and one foot compartment syndrome in 17 severe pilon fractures treated with a Taylor spatial frame [30].

Radiological evidence of secondary osteoarthrosis was seen in all but 8 of the 34 patients despite a good reduction in 12 patients. Poor definition of osteoarthrosis in previous studies has led to its under-reporting [2,9,17,23,31]. Guo et al., in a retrospective comparative study of 78 Ruedi type III fractures, treated either with ORIF or external fixation combined with limited internal fixation, reported a significantly higher incidence of traumatic arthritis in the external fixation group [32]. Wyrsch et al. found some degree of radiological osteoarthrosis in all 17 type III pilon fractures [20]. By randomizing patients into those that had internal fixation alone or combined internal-external fixation, they demonstrated that osteoarthrosis was independent of the type of treatment. Our conclusion, similar to theirs, is that the initiator of secondary osteoarthrosis is the severe initial articular damage that occurs at the time of injury. In our study, the severity of radiographic osteoarthrosis did not correspond with the end clinical result. Therefore, we suggest that devascularisation of the bone fragments during an open procedure, for an optimal anatomic reduction, could be a leading cause for the clinical manifestation and deterioration of osteoarthrosis. Furthermore, our observations that the quality of the reduction (T&W), the functional score (O&B), the subjective assessment (O&M), and the delay until surgery do not correlate with each other, further support the severity and the primary role of the initial injury pattern to the fracture’s outcome, prompting us to suggest that close reduction prevents the development of the clinical manifestations of osteoarthrosis.

The assessment, by the patients, of their own physical and mental health has been attempted before. In a group of low and high-energy pilon fractures treated by internal fixation, 40% of whom completed the 36-Item Short Form Health Survey (SF-36^TM^) health questionnaire [33], Sands et al. reported a significant reduction in the physical functioning components [34]. In our study, despite the majority obtaining good functional results with the Olerud and Molander ankle score [10], the patients’ perception of their injury, as assessed by the WHOQOL-BREF tool, was not so encouraging, with six patients scoring outside the normal population for the physical component. This puts the value of the functional scores in perspective and demonstrates the consequences of this serious injury on a previously normal active individual.

This study has several limitations. Firstly, it is a retrospective study without a control group, it includes only a small number of cases, although all of them are severe type III fractures, which is a specific type of injury, and there are not many studies available with higher patient numbers. Comparative studies of different kinds of treatment of this injury are also needed. The quality of reduction was measured with simple radiographs and not with a CT scan, which provides a better and three-dimensional view of the reduction of the fracture. We believe that the criteria proposed by Ovadia and Beals and modified by Teeny and Wiss can also be a good measuring tool for the quality of reduction.

## Conclusions

The method of treatment significantly affects the clinical outcome of pilon type III fractures. Complication rates can be reduced with olive tip wires and small fragment washers, early ligamentotaxis and fracture stabilization with the use of an Ilizarov ring fixator. These simple steps could lead safely to union and a good clinical and functional outcome.
